# Evaluation of diastolic dysfunction in children with hypertrophic cardiomyopathy and its relationship with development of myocardial fibrosis

**DOI:** 10.1186/s43044-023-00382-1

**Published:** 2023-06-30

**Authors:** Salem Elsayed Deraz, Omar Deyaa Esmat, Rehab Galal Abd El-Hmid, Sayed Ali Amin

**Affiliations:** 1 Pediatric Department, Menoufia University, Menoufia, Egypt; 2 Pediatric Cardiology Department, Aswan Heart Centre, Aswan, Egypt; 3 Pediatric Department, Fayoum University, Fayoum, Egypt

**Keywords:** Hypertrophic cardiomyopathy, Myocardial fibrosis, Diastolic dysfunction

## Abstract

**Background:**

Patients with hypertrophic cardiomyopathy may develop symptoms of shortness of breathing due to diastolic dysfunction which is not related to the severity of left ventricular outflow tract obstruction. As these patients usually develop a non-ischemic pattern of myocardial fibrosis, this may represent a mechanism for increased myocardial stiffness leading to impaired diastolic filling. The study aimed to determine the prevalence of myocardial fibrosis assessed by magnetic resonance imaging in children with hypertrophic cardiomyopathy and to evaluate its relationship with echocardiographic parameters including left ventricle diastolic dysfunction and to find echocardiographic indices which correlates with myocardial fibrosis as detected by cardiac magnetic resonance. A cross-sectional study was done for data of 50 children with hypertrophic cardiomyopathy from July 2018 to July 2021, patients were divided into (group 1) having myocardial fibrosis and (group 2) with no myocardial fibrosis, and results of echocardiographic parameters were compared between the two groups.

**Results:**

Results showed strong relationship between presence of myocardial fibrosis and each of the following: Interventricular septum thickness, lower lateral and septal early diastolic tissue velocities (*E*′), *E*/*E*′ ratio, presence of left ventricular out flow tract obstruction and the grade of diastolic dysfunction.

**Conclusions:**

The trans-mitral lateral and septal *E*/*E*′ (early mitral inflow to early diastolic mitral annular velocity ratio) allows early detection of left ventricular diastolic dysfunction in children with hypertrophic cardiomyopathy. The prevalence of diastolic dysfunction is higher in obstructive hypertrophic cardiomyopathy. The diastolic dysfunction severity is higher in patients with myocardial fibrosis.

## Background

Hypertrophic cardiomyopathy (HCM) is a relatively common genetic cardiac disease, accounting for 42% of childhood cardiomyopathy [1]. The severity of cardiac hypertrophy, etiology, as well as the clinical course of hypertrophic cardiomyopathy (HCM) in children, is varied, resulting in a large spectrum of clinical and phenotypic expression [2, 3]. Many patients develop symptoms of breathlessness due to diastolic dysfunction which is largely independent of the severity of left ventricular outflow tract obstruction (LVOTO) [4]. As patients with hypertrophic cardiomyopathy (HCM) frequently demonstrate a non-ischemic pattern of myocardial fibrosis, this may represent a mechanism for increased myocardial stiffness leading to impaired diastolic filling [5]. Magnetic resonance imaging (MRI) with late gadolinium enhancement (LGE) can detect a small and focal myocardial fibrosis [6].

Magnetic resonance imaging has been widely accepted for detection of myocardial fibrosis, which has been implicated as a factor in cardiovascular events and left ventricular (LV) function [7]. Echocardiographic modalities such as tissue Doppler imaging (TDI) have become a sensitive measure of diastolic ventricular dysfunction. They are relatively load independent and can reliably determine the degree of left ventricular diastolic dysfunction, which affects the clinical course in children with hypertrophic cardiomyopathy (HCM) [8].

### Aim of the study

The aim of this study is to determine the diastolic dysfunction in children having hypertrophic cardiomyopathy (HCM) by echocardiography modalities and to evaluate its relationship with prevalence of myocardial fibrosis as detected by cardiac magnetic resonance imaging and if possible to find echocardiographic indices which correlates with myocardial fibrosis as detected by cardiac magnetic resonance (CMR).

## Methods

The current study is a cross-sectional study which was done for data of 50 patients with hypertrophic cardiomyopathy (HCM) in pediatric age group (below age of 18 years). All cases were evaluated at Aswan Heart Centre, Aswan, Egypt.

### Background and demographic characteristics

*Inclusion criteria*: Age group: below age of 18 years, both sexes, echocardiographic evidence of myocardial hypertrophy defined as a diastolic septal thickness or left ventricular diastolic wall thickness z-score > 2. The MRI and echocardiography were done during the same visit.

*Exclusion criteria*: Presence of hemodynamic conditions that could account for the observed hypertrophy (as systemic hypertension), presence of congenital anomalies resulting in pressure overload against left ventricle (as congenital aortic stenosis or coarctation of aorta). Atrial fibrillation also was one of the exclusion criteria.

All patients were subjected to:

1. *Full history taking* focusing on patient demographics: age, sex, weight, height & body surface area, clinical symptoms of heart failure or low cardiac output (dyspnea on exertion, dizziness, syncope, chest pain), family history of hypertrophic cardiomyopathy (HCM), family history of sudden cardiac death (SCD) defined as one or more cardiac death in first-degree relatives of < 40 years of age with or without a diagnosis of hypertrophic cardiomyopathy (HCM) or sudden cardiac death (SCD) in first-degree relatives with confirmed hypertrophy at any age [9].
2. *Echocardiography* all echocardiograms were done by echo machine Philips IE33, Two-dimensional ECHO focusing on: Presence of hypertrophic cardiomyopathy was diagnosed when the diastolic septal thickness or left ventricular diastolic wall thickness z-score > 2 in the absence of hemodynamic conditions that could account for the observed LV hypertrophy [9], Left atrial (LA) diameter (mm, z-score) and left atrial volume indexed to body surface area. Presence of left ventricular outflow tract obstruction (LVOTO). Left ventricular outflow tract obstruction (LVOTO) was considered present when a peak outflow gradient of ≥ 30 mm Hg with continuous-wave Doppler echocardiography. Assessment of mitral regurgitation severity, according to relation of regurgitant area to left atrium area (< 15% is mild, 15–50% is moderate, > 50% is severe) [10]. Assessment of tricuspid regurgitation and estimation of the tricuspid regurge velocity by meter/sec. Conventional pulsed Doppler focusing on: mitral inflow pattern at the leaflet tips in the apical 4-chamber view, peak velocities of E (early mitral inflow) and A (late mitral inflow corresponds to atrial contraction) waves (cm/s) and their ratio (E/A) were measured in 3 successive cardiac cycles and average value of each measurement was taken. A wave duration by msec measured in 3 successive cardiac cycles was measured and average value was taken right upper pulmonary vein flow with measurement of atrial reversal flow (AR wave) duration in 3 successive cardiac cycles, and average value was taken (in patients with sinus rhythm). Tissue Doppler imaging (TDI) with breath holding during 3 consecutive cardiac cycles (whenever possible) focusing on: longitudinal annular velocities at the lateral as well as the septal mitral annular points. Early diastolic (*E*′) and late diastolic (*A*′) tissue Doppler velocities (cm/s) were measured at the lateral as well as the septal mitral annular points and subsequently averaged over 3 cardiac cycles. Trans-mitral *E*/*E*′ ratios (lateral and septal) were calculated for each patient. M-mode echocardiography focusing on septal wall thickness (SWT) and left ventricular posterior wall thickness (LVPWT) (mm, z-score).
  Diastolic dysfunction is assessed in patients with hypertrophic cardiomyopathy (HCM) as follows: 4 cut-off values for assessment of diastolic dysfunction are: Average *E*/*E*′ ratio > 14, left atrial (LA) volume indexed > 34 ml/m^2^, Atrial flow reversal (AR-A wave) duration > 30 ms, Peak velocity of tricuspid regurgitation (TR) jet by CW Doppler > 2.8 m/sec. In case of severe mitral regurge only 2 variables are applied which are AR-A duration and peak velocity of tricuspid regurgitation (TR) jet. Presence of more than half of the variables means that left atrial pressure (LAP) is elevated and grade II diastolic dysfunction is diagnosed; if less than half of the variables is present, then left atrial pressure (LAP) is normal and grade I diastolic dysfunction is diagnosed. Grade III diastolic dysfunction is diagnosed in the presence of restrictive filling pattern and abnormally reduced annular *E*′ velocity (septal < 7 cm/sec, lateral < 10 cm/sec). Absence of any of the variables means that no diastolic dysfunction [11]. Z score calculation was done by the following online calculator: http://parameterz.blogspot.com/2008/09/z-scores-of-cardiac-tructures.html.
3. *Cardiac magnetic resonance imaging (CMR)* Magnetic resonance imaging was performed on a 1.5 T scanner (Siemens, Germany). Cine images were acquired with steady-state free-precession technique (true FISP; slice thickness 8 mm) in three long-axis planes and contiguous short-axis slices (true FISP; slice thickness 8 mm, 2 mm gap) from the atrioventricular ring to the apex. An intravenous bolus of 0.1 mmol/kg of gadodiamide is given and late gadolinium images were acquired in the same planes after 10 min, with a breath-hold (whenever possible) segmented inversion-recovery sequence (inversion time: 280–400 ms). The presence of left ventricular late gadolinium enhancement (LGE) was determined using visual assessment by two independent observers.

### Statistical analysis

The collected data were organized, tabulated, and statistically analyzed using SPSS software statistical computer package version 18 SPSS Inc, USA. For quantitative data, the mean and standard deviation (SD) were calculated. Independent t-test as used in comparing between two groups as regards mean values of different variables. Qualitative data were presented as number and percentages; Chi-square (X^2^) was used as a test of significance. For interpretation of results of significance, significance was adopted at *P* < 0.05.

## Results

The current study aimed to determine the diastolic dysfunction in children having hypertrophic cardiomyopathy (HCM) by echocardiography modalities and to evaluate its relationship with prevalence of myocardial fibrosis as detected by cardiac magnetic resonance imaging. Based on the MRI findings, children were divided into two groups as follows: Group A: children diagnosed as having hypertrophic cardiomyopathy (HCM) with myocardial fibrosis. Group B: children having hypertrophic cardiomyopathy (HCM) without myocardial fibrosis. The results of echocardiographic parameters regarding presence or absence of diastolic dysfunction and its degree were analyzed and compared between the two groups.

**Fig. 1:**
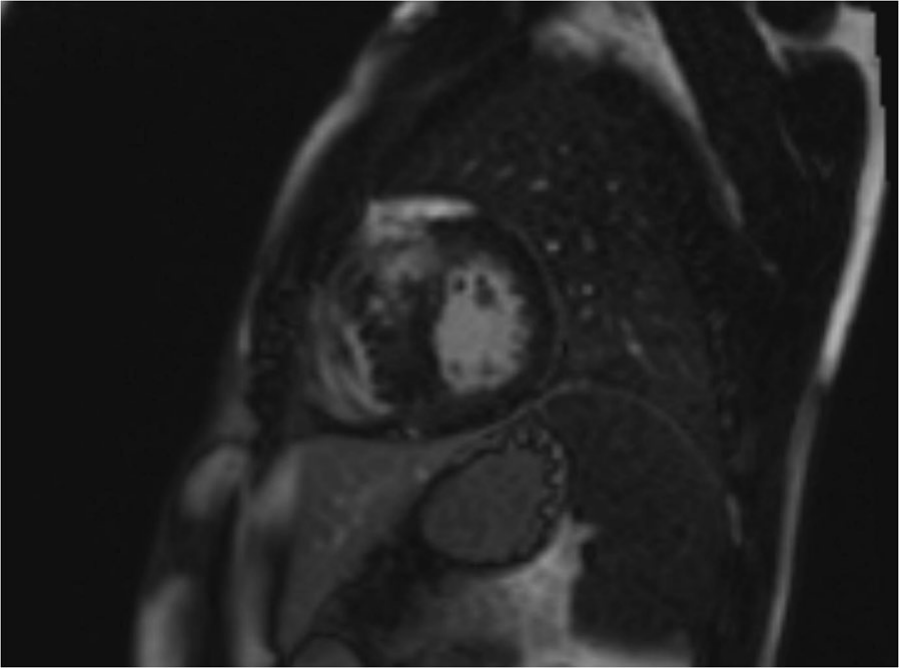
MRI short axis with delayed gadolinium enhancement

**Fig. 2:**
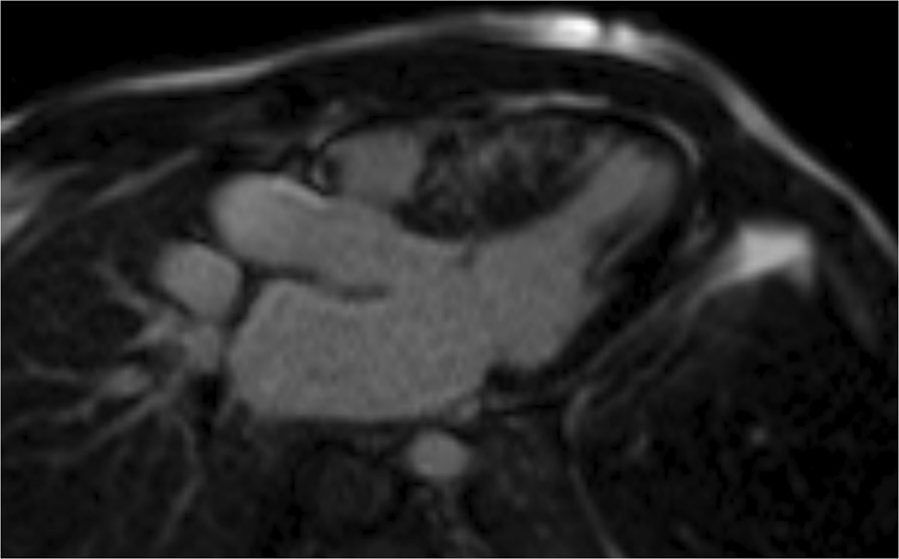
MRI 3 chamber view with delayed gadolinium enhancement. *In the 2 images there is mid-myocardial mural enhancement involving the asymmetrically hypertrophied interventricular septum*

The current study was conducted on 50 patients in pediatric age group with hypertrophic cardiomyopathy (HCM) from July 2018 to July 2021 (Table 1). We aimed to determine the diastolic dysfunction in children having hypertrophic cardiomyopathy (HCM) by echocardiography modalities and to evaluate its relationship with prevalence of myocardial fibrosis as detected by cardiac magnetic resonance imaging (Fig. 1 and 2).

**Table 1:** Socio-demographic characteristics of study participants

Variable	Mean	± SD
Age	8.44	± 4.21

Variable	*N*	%
Sex		
Male	36	72.0
Female	14	28.0

The mean age at Echo was 8.44 years; the mean age at MRI was 8.35 (Table 1). There was no time gap between Echo and MRI, both Echo and MRI were done during the same visit, 72% of cases were males (36 patients) and 28% of patients were females (14 patients). The mean body weight was 25.95 kg, the mean height was 119.71 cm and the mean BSA was 0.92. Our study showed that the mean SWTD Z-score was 5.49, mean LVPWD Z-score was 3.72 and mean left atrial (LA) dimension Z-score was 3.22 (Table 2), although Z-scores of all parameters were high, the thickness was marked in septal wall rather than posterior wall which translates the usual tendency of hypertrophy in hypertrophic cardiomyopathy (HCM) to be asymmetrical (Table 2).

**Table 2:** 2D echocardiography characteristics in studied patients

Parameters	Mean	± SD
SWTD (mm)	19.20	± 7.86
SWTD (z score)	5.49	± 1.53
LVPWD (mm)	10.59	± 3.89
LVPWD (z score)	3.72	± 1.52
LA dimensions (mm)	33.55	± 8.21
LA dimensions (z score)	3.22	± 1.41
LA volume index (ml/m^2^)	47.68	± 20.62

Among the 50 patients of the studied group, 84% of cases had a degree of mitral regurgitation (MR), which was caused mainly by systolic anterior motion (SAM) of mitral valve leaflets, 36% of the studied group had severe mitral regurgitation which affected cut-off points used in evaluation of diastolic dysfunction [11] (Table 3).

**Table 3:** Distribution of study participants regarding diastolic dysfunction grades

Variable	*N*	%
Diastolic dysfunction		
No diastolic dysfunction	8	16.0
Grade 1	6	12.0
Grade 2	21	42.0
Grade 3	15	30.0

Tissue Doppler imaging (TDI) parameters results showed mean values which meet cut-off values for diagnosis of diastolic dysfunction, mean velocity of lateral E` was 8.76 cm/s (cut-off value < 10 cm/s), mean lateral *E*/*E*′ was 13.74 (cut-off value > 14), mean medial E` velocity was 6.2 cm/s (cut-off value < 7 cm/s) and mean septal *E*/*E*′ was 18.57 (cut-off value > 14) as seen in Table 4. MRI-based presence of myocardial fibrosis was strongly correlated with tissue Doppler velocity indices especially septal *E*′ and (*E*/*E*′) ratio (Table 5).

**Table 4:** Different parameters of Pulsed Doppler and tissue Doppler in studied patients

Parameters	Mean	± SD
E velocity (cm/s)	92.22	± 25.91
A velocity (cm/s)	82.15	± 35.84
A duration (cm/sec)	131.58	± 30.97
AR duration (cm/sec)	160.14	± 28.84
AR-A	26.56	± 18.86
Lateral *E*′ (cm/s)	8.76	± 3.97
Lateral *A*′ (cm/s)	7.33	± 3.46
Lateral * E*/*E*′	13.74	± 10.89
Septal *E*′ (cm/s)	6.20	± 2.52
Septal *A*′ (cm/s)	5.56	± 1.84
septal * E*/*E*′	18.57	± 15.03

**Table 5:** Differences in 2D and TDI echocardiography characteristics according to fibrosis

Parameters	Patients with myocardial fibrosis (*N* = 22)		Patients without myocardial fibrosis (*N* = 28)		*P*-value
	Mean	± SD	Mean	± SD	
Lateral *E*′ (cm/s)	6.83	± 2.64	10.28	± 4.21	0.002*
Lateral *A*′ (cm/s)	7.21	± 2.59	7.42	± 4.06	0.833
Lateral * E*/*E*′	19.29	± 14.39	9.38	± 3.05	0.004*
Septal *E*′ (cm/s)	4.80	± 1.37	7.30	± 2.68	< 0.0001*
Septal *A*′ (cm/s)	5.43	± 1.66	5.66	± 1.99	0.654
Septal * E*/*E*′	25.66	± 19.75	13.00	± 5.79	0.008*
M-Mode measurements					
SWTD (mm)	23.08	± 8.58	16.15	± 5.73	0.001*
SWTD (z score)	6.10	± 1.48	5.00	± 1.40	0.010*
LVPWD (mm)	12.46	± 3.69	9.12	± 3.44	0.002*
LVPWD (z score)	4.18	± 1.80	3.36	± 1.17	0.073
LA dimensions (mm)	35.47	± 7.25	32.04	± 8.72	0.144
LA dimensions (z score)	3.44	± 1.15	3.04	± 1.59	0.327
LA volume index (ml/m^2^)	50.64	± 20.41	45.36	± 20.86	0.374

*Significant means* p*-value is ≤ 0.05

Studying relationship between myocardial fibrosis and diastolic dysfunction (Table 6) showed very strong relationship between degree of diastolic dysfunction and presence of fibrosis, among cases with myocardial fibrosis all cases have diastolic dysfunction grade 2 or 3, while patients without myocardial fibrosis did not have grade 3 diastolic dysfunction, while among cases with no myocardial fibrosis, prevalence of grade 3 diastolic dysfunction was 0%, which showed how grade of diastolic dysfunction and myocardial fibrosis are strongly linked.

**Table 6:** Relation between Diastolic dysfunction and myocardial fibrosis

Diastolic dysfunction	Patients with myocardial fibrosis (*N* = 22)		Patients without myocardial fibrosis (*N* = 28)		*P*-value
	*N*	%	*N*	%	
No diastolic dysfunction	0	0.0	8	28.6	< 0.0001*
Grade 1	0	0.0	6	21.4	
Grade 2	7	31.8	14	50.0	
Grade 3	15	68.2	0	0.0	

*Significant means* p*-value is ≤ 0.05

Data showed significant relationship between presence of hypertrophic obstructive cardiomyopathy (HOCM) and diastolic dysfunction (*P* value ≤ 0.001) (Table 7). Studying relationship between pattern of hypertrophic cardiomyopathy (HCM) and presence of myocardial fibrosis (Table 8) showed that 75% of cases with non-obstructive hypertrophic cardiomyopathy (HCM) were free of myocardial fibrosis, while only 47% of cases with hypertrophic obstructive cardiomyopathy (HOCM) were free of myocardial fibrosis.

**Table 7:** Relation of diastolic dysfunction to presence or absence of obstructive HCM

Diastolic dysfunction	Patients with Obstructive HCM (*N* = 34)		Patients with non-Obstructive HCM (*N* = 16)		*P*-value
	*N*	%	*N*	%	
No diastolic dysfunction	2	5.9	6	37.5	0.001*
Grade 1	2	5.9	4	25.0	
Grade 2	15	44.1	6	37.5	
Grade 3	15	44.1	0	0.0	

*Significant means* p*-value is ≤ 0.05

**Table 8:** Relation between pattern of HCM and fibrosis

Diastolic dysfunction	Patients with fibrosis (*N* = 22)		Patients without fibrosis (*N* = 28)		*P*-value
	*N*	%	*N*	%	
Obstructive HCM	18	52.9	4	25.0	0.063
Non-obstructive HCM	16	47.1	12	75.0	

## Discussion

To our knowledge, this is the youngest population in which diastolic dysfunction was studied in hypertrophic cardiomyopathy (HCM). We found that the percentage of diastolic dysfunction in our study was 84% (Table 3), the percentage is high when compared to Ziolkowska et al. [12] who found that percentage of diastolic dysfunction was 54%, but in their study percentage of HOCM to the studied population was 24%, while in our study percentage of HOCM to studied population was 68%, this can be explained from our study by understanding the relationship between pattern of hypertrophic cardiomyopathy (HCM) and prevalence of diastolic dysfunction, as we found that there is a very strong relationship between diastolic dysfunction and presence of obstructive form of hypertrophic cardiomyopathy (HCM) (*P*-value = 0.001) which can explain the cause of the large discrepancy between both studies in the prevalence of diastolic dysfunction.

In current study, 44% of the studied group had myocardial fibrosis (22 patients) in comparison with Ziolkowska et al. [12] who found that 60% of studied group had myocardial fibrosis. Putting in consideration that our study population mean age is lower than the previous study by 4 years, this can answer the question why the percentage can differ between both studies. As we found from observations, myocardial fibrosis tends to appear in older age, and it is most probably a progressively ongoing process.

Based on MRI findings, children were classified as either patients with myocardial fibrosis according to MRI (*n* = 22; 44%) or without fibrosis (*n* = 28; 56%). In adult patients with hypertrophic cardiomyopathy (HCM), the presence of fibrosis according to MRI correlated with left ventricular (LV) wall thickness and left atrial (LA) size [13, 14]. Similarly, one study was applied on children with hypertrophic cardiomyopathy (HCM) showed that children with myocardial fibrosis had significantly increased septum thickness, a higher left ventricular posterior wall (LVPWT), and significantly larger left atrial (LA) dimension and volume index on echocardiography compared to patients without fibrosis [12], we found similar results in our study as children in the studied group with myocardial fibrosis had a significantly thicker interventricular septum (*P* ≤ 0.0001), compared to patients without fibrosis (Table 5).

Our results showed that MRI-based presence of myocardial fibrosis was strongly correlated with tissue Doppler velocity indices especially septal *E*′ and (*E*/*E*′) ratio (Table 5), And these results are comparable with previous studies which studied relationship between diastolic dysfunction and myocardial fibrosis [15, 16]. Our results demonstrated that children with myocardial fibrosis in MRI had significantly lower lateral and septal early diastolic tissue velocities (*E*′) as well as a significantly higher lateral and septal *E*/*E*′ ratio compared to patients without fibrosis, Ziolkowska et al. [12] reported similar findings in pediatrics, and Ellims et al. [17] reported similar findings in adults. Studying relationship between myocardial fibrosis and diastolic dysfunction (Table 6) showed very strong relationship between degree of diastolic dysfunction and presence of fibrosis, among cases with myocardial fibrosis all cases have diastolic dysfunction grade 2 or 3, while patients without myocardial fibrosis did not have grade 3 diastolic dysfunction, while among cases with no myocardial fibrosis, prevalence of grade 3 diastolic dysfunction was 0%, which showed how grade of diastolic dysfunction and myocardial fibrosis are strongly linked. These results are comparable with Moreo et al. [16], who found a very strong relationship between degree of diastolic dysfunction and degree of myocardial fibrosis in adults.

Our results showed significant relationship between presence of hypertrophic obstructive cardiomyopathy (HOCM) and diastolic dysfunction, (Table 7). Our data also showed that 75% of cases with non-obstructive hypertrophic cardiomyopathy (HCM) were free of myocardial fibrosis, while only 47% of cases with hypertrophic obstructive cardiomyopathy (HOCM) were free of myocardial fibrosis, (Table 8).

### Study limitations

In our study we faced some limitations as studying diastolic dysfunction in young (sedated) or older non co-operative children was difficult as its optimally done with breath holding during 3 consecutive cardiac cycles which is practically difficult. Similarly young (sedated) and older non co-operative patients with latent left ventricular outflow tract obstruction (LVOTO) which isn’t diagnosed by resting Echocardiography were missed as they couldn’t do exercise or Valsalva maneuver. Also there was high risk of anesthesia in patients with both severe left ventricular outflow tract obstruction (LVOTO) and severe right ventricular outflow tract obstruction (RVOTO), and in patients with impaired LV systolic and these patients couldn’t be studied by MRI, and hence were excluded, and being expensive, MRI couldn’t be repeated in minority of cases when its relatively old.

## Conclusions

Myocardial fibrosis in children with hypertrophic cardiomyopathy was associated with markers for disease severity such as greater septum thickness and impairment of left ventricular diastolic function. So, the trans-mitral lateral and septal *E*/*E*′ (early mitral inflow to early diastolic mitral annular velocity ratio) together with measurement of inter-ventricular septal thickness allow early detection of myocardial fibrosis in children with hypertrophic cardiomyopathy. Echocardiography with tissue Doppler imaging is a feasible and sensitive method for evaluating left ventricular diastolic dysfunction in children with hypertrophic cardiomyopathy and myocardial fibrosis. Pediatric patients with obstructive form of hypertrophic cardiomyopathy have higher incidence (and high grade when present) of diastolic dysfunction that non-obstructive form. Higher degrees of diastolic dysfunction are higher in patients with myocardial fibrosis who always have at least 2nd degree of diastolic dysfunction, while patients without myocardial fibrosis never had grade 3 diastolic dysfunction.

## Data Availability

Authors declare availability of data used in the study.

## Abbreviations

HCM: Hypertrophic cardiomyopathy
LVOTO: Left ventricular outflow tract obstruction.
MRI: Magnetic resonance imaging
LGE: Late gadolinium enhancement
TDI: Tissue Doppler imaging
CMR: Cardiac magnetic resonance
SCD: Sudden cardiac death
AR: Atrial reversal
SWT: Septal wall thickness
LVPWT: Left ventricular posterior wall thickness.
LAP: Left atrial pressure
LA: Left atrium
MR: Mitral regurgitation
SAM: Systolic anterior motion
